# Immediate Spirometric Response to Submaximal Exercise in Healthy Young Adult Males

**DOI:** 10.7759/cureus.110381

**Published:** 2026-06-07

**Authors:** Ashwani Saharan, Vishavdeep Kaur, Ankalayya Bobbara

**Affiliations:** 1 Department of Physiology, Maharishi Markandeshwar Institute of Medical Sciences and Research, Ambala, IND

**Keywords:** acute exercise, harvard step test, pulmonary function, spirometry, submaximal exercise

## Abstract

Background: Acute exercise produces immediate changes in respiratory mechanics and airflow, but the pattern of spirometric response after a single bout of submaximal exercise is not uniform across studies. This study evaluated the immediate effect of a single bout of submaximal exercise on spirometric parameters in healthy young adult males.

Methodology: This prospective pre-post interventional study was conducted in the department of physiology over two years. A total of 200 healthy young adult males aged 18-30 years were included. Participants with a history of smoking, respiratory or cardiovascular disease, bronchial asthma, allergy, recent acute illness, alcoholism, athletic training, or musculoskeletal limitation were excluded. Baseline anthropometry was recorded. Spirometry was performed before exercise using a standard technique, with a minimum of three acceptable maneuvers obtained for each participant. Participants then underwent a single bout of submaximal exercise using the Harvard Step Test with a 16-inch step at a cadence of 40 steps per minute for six minutes. Spirometry was repeated immediately after exercise. Forced vital capacity (FVC), forced expiratory volume in one second (FEV1), peak expiratory flow rate (PEFR), and FEV1/FVC ratio were analyzed. Pre- and post-exercise values were compared using the Wilcoxon signed-rank test; p < 0.05 was considered statistically significant.

Results: The mean age of the participants was 22.12 ± 3.72 years. The mean FVC decreased significantly from 3.77 ± 0.82 L to 3.54 ± 0.74 L (p = 0.001). The mean FEV1 increased significantly from 3.26 ± 0.54 L to 3.41 ± 0.63 L (p = 0.020). PEFR also increased significantly from 7.26 ± 2.28 to 9.36 ± 1.70 (p < 0.001). The FEV1/FVC ratio changed from 0.84 ± 0.08 to 0.85 ± 0.08, which was not statistically significant (p = 0.337).

Conclusions: A single bout of submaximal exercise produced significant immediate changes in spirometric parameters in healthy young adult males. Expiratory flow indices improved after exercise, while FVC decreased, and the FEV1/FVC ratio remained stable.

## Introduction

Pulmonary function testing, particularly spirometry, remains a standard objective method for assessing lung volumes and expiratory airflow, and it is useful for evaluating both baseline respiratory status and short-term physiological responses to interventions such as exercise [1,2].

In lung-healthy populations, habitual physical activity has generally been associated with slightly better spirometric performance or slower decline in lung function [2-4]. However, the strength of this association is usually modest rather than large [3-5].

Acute exercise can also immediately modify respiratory physiology by altering ventilatory demand, airway caliber, and respiratory mechanics [2,6]. Recent studies in healthy subjects have shown that post-exercise bronchodilation and favorable mechanical changes may occur even in the absence of respiratory disease, with reports of increased post-exercise forced expiratory volume in one second (FEV1) and reduced respiratory system impedance after submaximal activity [6-9].

The immediate spirometric response to exercise has not been uniform across studies. Some investigations have shown little or no significant short-term change in forced vital capacity (FVC), FEV1, the FEV1/FVC ratio, and peak expiratory flow rate (PEFR) after acute exercise. In contrast, others have demonstrated improvement in selected airflow indices or protocol-dependent differences in the magnitude of response [8-11]. This variation is likely related to differences in exercise modality, exercise intensity, timing of post-exercise measurements, and the characteristics of the study population [6-8]. Healthy young adult males provide a relatively homogeneous group for studying the immediate physiological response to submaximal exercise because major confounders such as chronic cardiorespiratory disease, smoking-related airflow limitation, and age-related decline in lung function are minimized [1,7].

The present study was undertaken to evaluate the immediate effect of a single bout of submaximal exercise on spirometric parameters in healthy young adult males. Specifically, FVC, FEV1, PEFR, and the FEV1/FVC ratio were compared before and after exercise.

## Materials and methods

Study design and setting

This prospective pre-post interventional study was conducted in the department of physiology over a period of two years. The study was designed to assess the immediate effect of a single bout of submaximal exercise on pulmonary function in healthy young adult males.

Study population

The study included 200 healthy young adult males aged 18-30 years. Participants were recruited from the eligible study population after screening according to predefined inclusion and exclusion criteria. Only apparently healthy males without known respiratory or cardiovascular disease were included. Individuals with a history of smoking, bronchial asthma, allergy, alcoholism, recent acute illness or fever within the preceding two weeks, regular bronchodilator use, athletic training, or musculoskeletal injury limiting exercise capacity were excluded. Written informed consent was obtained from all participants before enrollment.

Baseline assessment

Baseline demographic and anthropometric data were recorded for all participants. Age was documented, height was measured using a calibrated stadiometer, and weight was measured using a calibrated weighing scale. Body mass index (BMI) was calculated as weight in kilograms divided by the square of height in meters [12].

Spirometry

Baseline pulmonary function testing was performed before exercise using a calibrated spirometer according to the standardized recommendations of the American Thoracic Society and European Respiratory Society [13]. Participants were instructed regarding the correct forced expiratory maneuver. A minimum of three technically acceptable maneuvers was obtained from each participant. Maneuvers were assessed according to standard acceptability and repeatability criteria, and the best values from acceptable and repeatable curves were used for analysis [13]. The spirometric parameters recorded were FVC, FEV1, PEFR, and FEV1/FVC ratio.

Exercise protocol

After baseline spirometry, all participants performed a single bout of submaximal exercise using the Harvard Step Test, as described by Gallagher and Brouha [14]. The Harvard Step Test is a non-proprietary, freely available physical fitness assessment tool commonly used in physiological and exercise-based research. A 16-inch platform was used, and the stepping cadence was maintained at 40 steps per minute. The total exercise duration was six minutes. The same protocol was used for all participants.

Post-exercise spirometry

Immediately after completion of the exercise protocol, spirometry was repeated using the same instrument and procedure as used for baseline assessment [13]. Post-exercise values of FVC, FEV1, PEFR, and FEV1/FVC ratio were recorded for comparison with pre-exercise values.

Outcome measures

The primary outcome measures were the immediate pre- to post-exercise changes in FVC, FEV1, PEFR, and FEV1/FVC ratio.

Statistical analysis

Data were entered and analyzed using Jamovi software [15]. Continuous variables were summarized as mean ± standard deviation. Absolute and percentage changes in spirometric parameters were also described where relevant. Pre- and post-exercise comparisons were performed using the Wilcoxon signed-rank test. A p-value of less than 0.05 was considered statistically significant.

## Results

A total of 200 healthy young adult males were included in the study. The mean age of the participants was 22.12 ± 3.72 years. The mean height was 1.75 ± 0.08 m, the mean weight was 66.18 ± 7.34 kg, and the mean body mass index was 21.67 ± 3.13 kg/m², as shown in Table 1.

**Table 1:** Baseline characteristics of the study participants.

Variable	n	Mean ± SD
Age (years)	200	22.12 ± 3.72
Height (m)	200	1.75 ± 0.08
Weight (kg)	200	66.18 ± 7.34
Body mass index (kg/m²)	200	21.67 ± 3.13

Comparison of spirometric parameters before and after acute submaximal exercise is shown in Table 2. The mean FVC decreased significantly from 3.77 ± 0.82 L before exercise to 3.54 ± 0.74 L after exercise (Z = -3.298, p = 0.001). The mean FEV1 increased significantly from 3.26 ± 0.54 L to 3.41 ± 0.63 L (Z = -2.332, p = 0.020). The mean PEFR also increased significantly from 7.26 ± 2.28 to 9.36 ± 1.70 (Z = -8.732, p < 0.001). The FEV1/FVC ratio showed a small increase from 0.84 ± 0.08 to 0.85 ± 0.08, but this difference was not statistically significant (Z = -0.961, p = 0.337).

**Table 2:** Comparison of spirometric parameters before and after acute submaximal exercise.

Parameter	Pre-exercise, Mean ± SD	Post-exercise, Mean ± SD	Test statistic	p-value
Forced vital capacity (FVC) (L)	3.77 ± 0.82	3.54 ± 0.74	Z = -3.298	0.001
Forced expiratory volume in 1 second (FEV1) (L)	3.26 ± 0.54	3.41 ± 0.63	Z = -2.332	0.020
Peak expiratory flow rate (PEFR)	7.26 ± 2.28	9.36 ± 1.70	Z = -8.732	<0.001
FEV1/FVC ratio	0.84 ± 0.08	0.85 ± 0.08	Z = -0.961	0.337

The absolute change in spirometric parameters after exercise is presented in Table 3. The mean change in FVC was -0.24 ± 1.15 L, with a median change of -0.21 L (IQR: -1.04 to 0.24). The mean change in FEV1 was 0.15 ± 0.78 L, with a median change of 0.10 L (IQR: -0.37 to 0.71). PEFR showed the largest absolute rise, with a mean change of 2.10 ± 2.81 and a median change of 1.50 (IQR: 0.23 to 4.06). The mean change in the FEV1/FVC ratio was 0.01 ± 0.11, with a median change of 0.01 (IQR: -0.05 to 0.07).

**Table 3:** Absolute change in spirometric parameters after acute submaximal exercise. FVC: forced vital capacity; FEV1: forced expiratory volume in one second; PEFR: peak expiratory flow rate.

Parameter	Mean change ± SD	Median (IQR)
Change in FVC (L)	-0.24 ± 1.15	-0.21 (-1.04 to 0.24)
Change in FEV1 (L)	0.15 ± 0.78	0.10 (-0.37 to 0.71)
Change in PEFR	2.10 ± 2.81	1.50 (0.23 to 4.06)
Change in FEV1/FVC ratio	0.01 ± 0.11	0.01 (-0.05 to 0.07)

The percentage change in spirometric parameters is shown in Table 4. FVC showed a mean percentage change of -1.16% ± 33.32%, with a median percentage change of -5.64%. FEV1 showed a mean percentage increase of 7.31% ± 26.09%, with a median increase of 2.89%. PEFR demonstrated the highest percentage rise, with a mean change of 45.38% ± 63.75% and a median change of 19.16%. The FEV1/FVC ratio showed only a small mean percentage increase of 1.78% ± 13.38%, with a median change of 0.91%.

**Table 4:** Percentage change in spirometric parameters after acute submaximal exercise. FVC: forced vital capacity; FEV1: forced expiratory volume in one second; PEFR: peak expiratory flow rate.

Parameter	Mean % change ± SD	Median % change	Minimum % change	Maximum % change
FVC	-1.16 ± 33.32	-5.64	-47.87	133.91
FEV1	7.31 ± 26.09	2.89	-38.19	81.33
PEFR	45.38 ± 63.75	19.16	-37.01	256.51
FEV1/FVC ratio	1.78 ± 13.38	0.91	-26.32	38.03

## Discussion

The present study evaluated the immediate effect of a single bout of submaximal exercise on spirometric parameters in healthy young adult males. As shown in Table 1, the study population was relatively homogeneous, with a mean age of 22.12 ± 3.72 years and a mean body mass index of 21.67 ± 3.13 kg/m². This homogeneity is important because the findings can be interpreted with less confounding from advanced age, smoking, or chronic cardiorespiratory disease. In Table 2, acute exercise was followed by a significant decrease in FVC, a significant increase in FEV1, a marked increase in PEFR, and no significant change in the FEV1/FVC ratio. This overall pattern suggests that a brief bout of submaximal exercise may acutely improve expiratory flow more than static expiratory volume in healthy young men [6,8].

The reduction in FVC observed in Table 2, from 3.77 ± 0.82 L to 3.54 ± 0.74 L, is notable because it does not follow the common expectation that exercise uniformly improves all spirometric indices. Some studies [10,16] have reported no significant post-exercise change in spirometric indices in healthy adults. One possible explanation is that acute exercise places a transient load on the ventilatory system and may alter breathing mechanics, expiratory effort, or end-expiratory operating volume immediately after exertion [2]. Furquim et al. [6] also demonstrated that after submaximal exercise in 50 healthy adults, respiratory impedance fell immediately after exertion, suggesting that acute exercise modifies airway mechanics in a manner that may favor airflow but not necessarily maximal exhaled volume. This may partly explain why FVC declined while FEV1 and PEFR improved in the present study.

In contrast, FEV1 increased significantly in Table 2, from 3.26 ± 0.54 L to 3.41 ± 0.63 L. This finding is physiologically plausible and may reflect transient exercise-induced bronchodilation, reduced airway resistance, and improved expiratory flow immediately after exertion [8,9]. Welch et al. [8] reported that exercise-induced bronchodilation one minute after exercise was present in 54% of younger adults and 64% of older adults, compared with only 15% of adolescents. Similarly, Thompson et al. [9] observed post-exercise improvement in FEV1 in both sexes, with FEV1 increasing from 4.80 ± 0.63 L to 5.03 ± 0.58 L in males and from 3.81 ± 0.31 L to 3.96 ± 0.32 L in females. Therefore, the increase in FEV1 in the present study is consistent with the concept that acute whole-body exercise can transiently dilate the airways in healthy individuals [8,9].

The largest acute response in the present study was observed for PEFR. In Table 2, PEFR increased markedly after exercise, while Table 3 showed a mean absolute increase of 2.10 ± 2.81, and Table 4 demonstrated the largest mean percentage rise among all measured parameters at 45.38% ± 63.75%. This likely indicates that acute exercise had a greater effect on early force-dependent expiratory flow than on total exhaled volume [6,9]. Although direct comparisons across studies are difficult because of differences in exercise protocols and the timing of post-exercise spirometry, Angane and Navare [17] reported improvements in expiratory flow after aerobic training in 65 healthy adults, with maximum forced expiratory flow (FEFmax) increasing from 7.72 to 9.22 L/s. Furquim et al. [6] also reported reduced post-exercise respiratory impedance following the six-minute walk test, further supporting the possibility of improved airway conductance after submaximal exertion. Together, these observations support the finding that airflow-related variables may demonstrate a more prominent acute response than volume-based variables.

Despite the opposite directional changes in FVC and FEV1, the FEV1/FVC ratio did not change significantly in the present study (Table 2). The absolute change shown in Table 3 and the minimal percentage change in Table 4 further suggest the relative stability of this ratio. Similar findings have been reported in other acute exercise studies involving healthy subjects [10,11]. Angane and Navare [17] also reported no significant change in the FEV1/FVC ratio after aerobic training, with the ratio remaining essentially unchanged from 84.98 before training to 84.38 after training. Likewise, Roopashree et al. [10] found no significant post-exercise change in FEV1/FVC in either normal or anemic young adults after bicycle exercise testing. However, findings across studies are not entirely uniform, with some reporting minimal or no change in healthy individuals [10,11].

The variability of response observed in the present study is also important. As shown in Table 4, percentage changes were widely distributed for all parameters, particularly for PEFR and FVC. This suggests that the acute spirometric response to exercise is not uniform, even among apparently healthy individuals. Previous studies have similarly reported inconsistent findings, with some demonstrating improvement in spirometric values and others showing little change or even reductions, depending on the exercise protocol, environment, and timing of measurement [18,19]. Jin et al. [18], in a crossover study involving 30 healthy young adults exposed to different pollution environments during 90 minutes of moderate-intensity exercise, observed increases in lung function in low-pollution settings but declines in FVC, FEV1, and PEFR in high-pollution settings.

In contrast, Kim et al. [19] studied nine healthy young men and found no significant differences in FVC, FEV1, or FEV1/FVC after acute moderate-intensity aerobic exercise under high versus low PM2.5 conditions. Similarly, Gowhar et al. [11] reported no significant overall change in respiratory parameters after a six-minute Harvard Step Test in 90 MBBS students; however, overweight and obese females demonstrated a reduction in FVC and vital capacity (VC). These differences reinforce the concept that immediate post-exercise spirometry is influenced by the exercise model, participant characteristics, and the context in which testing is performed [10,18,19].

The present findings should be interpreted as an acute physiological response rather than a training effect. In contrast to the immediate post-exercise changes observed in the current study, structured exercise programs generally report broader improvements in spirometric indices over time. Rawashdeh et al. [20] reported significant improvements in FVC, FEV1, MVV, and FEV1/FVC following high-intensity aerobic training in 72 healthy inactive males. Similarly, Novotová et al. [21] reported that most walking-based interventions in healthy older adults were associated with improvements in lung function. In contrast, the present study reflects the immediate response to a single bout of step-test exercise rather than the adaptive effects of repeated exercise training.

The findings of the present study have potential clinical and research implications. Since significant changes in spirometric parameters were observed immediately after submaximal exercise, clinicians and researchers should consider recent physical activity when interpreting pulmonary function tests. Acute exercise may transiently enhance expiratory flow measurements such as FEV1 and PEFR while affecting volume-based parameters differently. Awareness of these physiological responses may help avoid misinterpretation of spirometric results and contribute to a better understanding of normal respiratory adaptations to exercise.

This study has several limitations. It was restricted to healthy young adult males; therefore, the findings cannot be generalized to females, older adults, athletes, or individuals with respiratory disease. Only immediate post-exercise measurements were obtained, and serial recovery spirometry could have clarified whether the observed pattern was transient or sustained. Additionally, spirometric parameters such as FVC and PEFR are effort-dependent measurements, and the influence of post-exercise fatigue on participant performance cannot be completely excluded. Furthermore, the study assessed only the immediate post-exercise response and therefore cannot determine the duration or persistence of the observed changes. In addition, repeated pulmonary function testing itself may influence performance over time, as demonstrated by Krabbe et al. [22], who reported a measurable training effect with repeated spirometry in healthy volunteers. The study also relied solely on conventional spirometric variables. Recent work by Stavarache et al. [23] suggests that small-airway and resistance-based techniques may detect respiratory changes after aerobic training that are not fully captured by routine spirometry.

The present study demonstrated that a single bout of submaximal exercise produced significant immediate changes in pulmonary function in healthy young adult males. FEV1 and PEFR increased, FVC decreased, and the FEV1/FVC ratio remained unchanged. The dominant acute effect appeared to be an improvement in expiratory flow rather than an increase in exhaled volume. These findings add to the existing evidence that the pulmonary response to acute exercise in healthy individuals is real but variable, and that different spirometric indices may change in different directions during the immediate post-exercise period.

## Conclusions

A single bout of submaximal exercise produced significant immediate changes in spirometric parameters in healthy young adult males. FEV1 and PEFR increased significantly after exercise, whereas FVC decreased, and the FEV1/FVC ratio remained unchanged. These findings suggest that the immediate respiratory response to submaximal exercise is characterized more by improvements in expiratory airflow than by increases in lung volume. Further studies with serial post-exercise measurements and the inclusion of additional physiological parameters are needed to better define the time course and mechanisms of these changes.

## References

[1] Dominelli PB, Sheel AW (2024). The pulmonary physiology of exercise. Adv Physiol Educ.

[2] Phillips DB, Stickland MK (2019). Respiratory limitations to exercise in health: a brief review. Curr Opin Physiol.

[3] Luzak A, Karrasch S, Thorand B, Nowak D, Holle R, Peters A, Schulz H (2017). Association of physical activity with lung function in lung-healthy German adults: results from the KORA FF4 study. BMC Pulm Med.

[4] Collaud S, Touilloux B, von Garnier C, Marques-Vidal P, Kraege V (2024). Physical activity and lung function association in a healthy community-dwelling European population. BMC Pulm Med.

[5] Puente-Maestu L, Stringer WW (2018). Physical activity to improve health: do not forget that the lungs benefit too. Eur Respir J.

[6] Furquim TH, Santos DO, Perossi J, Lima FC, Silveira JM, Gastaldi AC (2025). Acute effect of submaximal exercise on respiratory system impedance in healthy adults. J Funct Morphol Kinesiol.

[7] Satia I, Priel E, Al-Khazraji BK, Jones G, Freitag A, O'Byrne PM, Killian KJ (2021). Exercise-induced bronchoconstriction and bronchodilation: investigating the effects of age, sex, airflow limitation and FEV1. Eur Respir J.

[8] Welch R, Lardenoye M, Kolbe J, Ellyett K (2022). Exercise induced bronchodilation: a phenomenon more common, greater magnitude and more prolonged in older adults than in adolescents. J Asthma.

[9] Thompson BP, Doherty CJ, Dominelli PB (2025). Exercise-induced bronchodilation is not different in healthy males and females. J Appl Physiol.

[10] Roopashree K, John AJ, Dilip KD, Ragothaman PS, Krishna A (2025). Effect of acute exercise on pulmonary function in young adults with anaemia: a quasi-experimental study. J Clin Diagn Res.

[11] Gowhar M, Irum J (2019). A study on effect of acute exercise on pulmonary function tests of first year M.B.B.S. students. Int J Physiol.

[12] (2024). WHO Expert Committee on Physical Status. The use and interpretation of anthropometry. World Health Organ Tech Rep Ser.

[13] Graham BL, Steenbruggen I, Miller MR (2019). Standardization of spirometry 2019 update. An official American Thoracic Society and European Respiratory Society technical statement. Am J Respir Crit Care Med.

[14] Gallagher JR, Brouha L (1943). A simple method of evaluating fitness in boys: the step test. Yale J Biol Med.

[15] (2025). The Jamovi Project. Jamovi (version 2.3). https://www.jamovi.org/.

[16] Gavrielatos A, Ratkevica I, Stenfors N, Hanstock HG (2022). Influence of exercise duration on respiratory function and systemic immunity among healthy, endurance-trained participants exercising in sub-zero conditions. Respir Res.

[17] Angane E, Navare A (2016). Effects of aerobic exercise on pulmonary function tests in healthy adults. Int J Res Med Sci.

[18] Jin X, Wang W, Sun Q, Chen Y, Xu B, Tian H (2024). Effects of physical exercise on cardio-respiratory health of young adults during short-term exposure to varying air pollution levels. BMC Public Health.

[19] Kim JS, Lee DG, Wang L, Kang H, Hwang MH (2022). Acute moderate-intensity aerobic exercise under high PM2.5 levels does not influence the pulmonary function and lung diffusion capacity in healthy young men. Appl Sci.

[20] Rawashdeh A, Alnawaiseh N (2018). The effect of high-intensity aerobic exercise on the pulmonary function among inactive male individuals. Biomed Pharmacol J.

[21] Novotová K, Pavlů D, Dvořáčková D, Arnal-Gómez A, Espí-López GV (2022). Influence of walking as physiological training to improve respiratory parameters in the elderly population. Int J Environ Res Public Health.

[22] Krabbe J, Kotro AK, Kraus T (2023). Effects of repetition as training and incentives on the performance in pulmonary function tests in healthy volunteers. Heliyon.

[23] Stavarache IE, Cernomaz TA, Grosu-Creangă IA, Trofor A (2025). The effect of aerobic training on healthy small airways—a forced oscillation technique approach to optimize long term care in COPD. J Clin Med.

